# Time-dependent cell-state selection identifies transiently expressed genes regulating ILC2 activation

**DOI:** 10.1038/s42003-023-05297-w

**Published:** 2023-09-06

**Authors:** Yumiko Tanaka, Mai Yamagishi, Yasutaka Motomura, Takashi Kamatani, Yusuke Oguchi, Nobutake Suzuki, Tsuyoshi Kiniwa, Hiroki Kabata, Misato Irie, Tatsuhiko Tsunoda, Fuyuki Miya, Keisuke Goda, Osamu Ohara, Takashi Funatsu, Koichi Fukunaga, Kazuyo Moro, Sotaro Uemura, Yoshitaka Shirasaki

**Affiliations:** 1 Department of Biological Sciences, Graduate School of Science, The University of Tokyo, Tokyo, Japan; 2 Graduate School of Pharmaceutical Sciences, The University of Tokyo, Tokyo, Japan; 3 Live Cell Diagnosis, Ltd, Saitama, Japan; 4 Department of Microbiology and Immunology, Graduate School of Medicine, Osaka University, Osaka, Japan; 5 Department of AI Technology Development, M&D Data Science Center, Tokyo Medical and Dental University, Tokyo, Japan; 6 Division of Precision Cancer Medicine, Tokyo Medical and Dental University Hospital, Tokyo, Japan; 7 Division of Pulmonary Medicine, Department of Medicine, Keio University School of Medicine, Tokyo, Japan; 8 PRESTO, JST, Saitama, Japan; 9 RIKEN Cluster for Pioneering Research, Saitama, Japan; 10 RIKEN Center for Integrative Medical Sciences, Kanagawa, Japan; 11 Department of Computational Biology and Medical Sciences, Graduate School of Frontier Sciences, The University of Tokyo, Tokyo, Japan; 12 Center for Medical Genetics, Keio University School of Medicine, Tokyo, Japan; 13 Department of Chemistry, Graduate School of Science, The University of Tokyo, Tokyo, Japan; 14 Department of Bioengineering, University of California, Los Angeles, CA 90095 USA; 15 Institute of Technological Sciences, Wuhan University, Hubei, 430072 China; 16 Kazusa DNA Research Institute, Chiba, Japan

**Keywords:** Immune cell isolation, Time-lapse imaging, ELISPOT

## Abstract

The decision of whether cells are activated or not is controlled through dynamic intracellular molecular networks. However, the low population of cells during the transition state of activation renders the analysis of the transcriptome of this state technically challenging. To address this issue, we have developed the Time-Dependent Cell-State Selection (TDCSS) technique, which employs live-cell imaging of secretion activity to detect an index of the transition state, followed by the simultaneous recovery of indexed cells for subsequent transcriptome analysis. In this study, we used the TDCSS technique to investigate the transition state of group 2 innate lymphoid cells (ILC2s) activation, which is indexed by the onset of interleukin (IL)-13 secretion. The TDCSS approach allowed us to identify time-dependent genes, including transiently induced genes (TIGs). Our findings of *IL4* and *MIR155HG* as TIGs have shown a regulatory function in ILC2s activation.

## Abstract (web summary)

Time-Dependent Cell-State Selection combines live cell imaging and single-cell RNA sequencing to characterize and analyse ILC2s during their activation, revealing a set of genes that are transitionally upregulated (TIGs) in ILC2s during this phase.

## Introduction

**Fig. 1:**
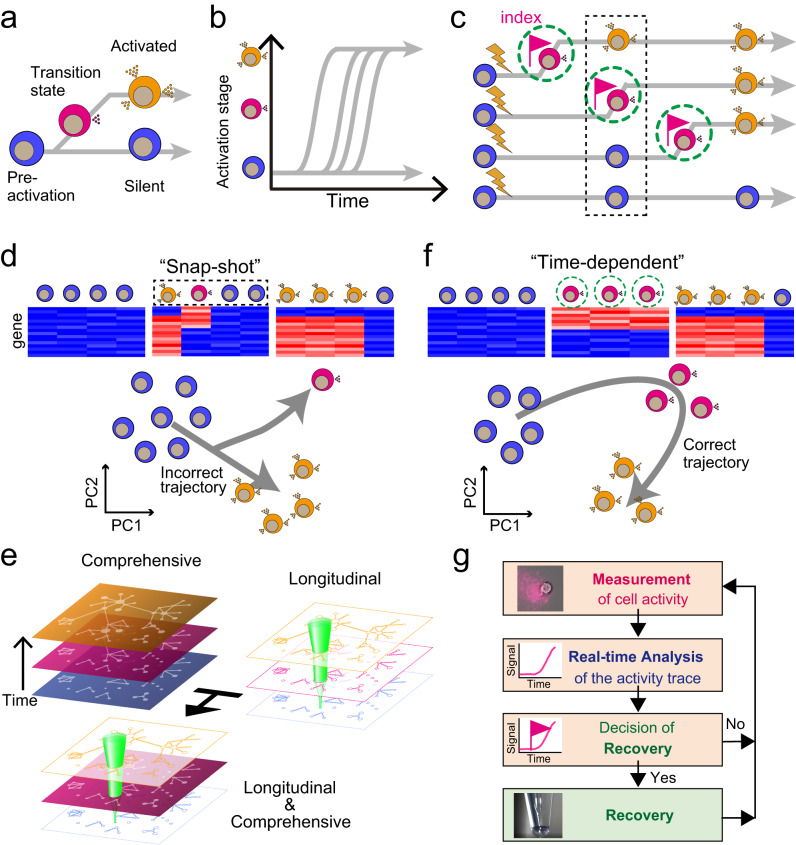
Concept of time-dependent cell-state selection, TDCSS. **a** Model depicting the transition of cellular states during the process of cell fate determination. **b** Variation in the timing of fate determination among cells. **c** Comparison of two cell recovery methods during the process of cell fate determination. The conventional strategy involves collecting cells in a “snapshot” manner (black dashed rectangle), while the “time-dependent” method involves collecting cells at each cell’s individual transition timing (green dashed circle). **d** Single-cell transcriptomes obtained through snapshot cell recovery methods and inferred trajectories of fate determination. **e** TDCSS methodology combines longitudinal and comprehensive analysis. **f** Single-cell transcriptomes obtained through time-dependent cell recovery methods and deduced trajectories of fate determination. **g** Schematic representation of the TDCSS technique.

Cells determine their fate, such as differentiation^1^, cell death^2^, and activation during stress response^3^ and immune response^4^, through dynamic intracellular molecular mechanisms^5,6^. In order to comprehend the mechanisms by which cells determine their fate, it is necessary to identify the molecular signatures of the early stages of the fate determination process, which control gene expression and ultimately determine the cell’s fate (Fig. 1a). In general, as fate determination is asynchronous^7–9^ (Fig. 1b), a population of cells collected by a “snap-shot” manner at a specific time point would contain cells in various stages of the fate determination process (Fig. 1c, d).

There are two traditional methods for studying cell fate determination processes. Single-cell transcriptome analysis allows us to computationally infer the fate determination process by ordering cells based on similarities in their gene expression patterns^10,11^ (Fig. 1d). This approach is effective for slow fate determination processes, such as differentiation, but it is not effective for rapid fate determination processes with a short transition period, such as inflammation. Additionally, this approach can sometimes inaccurately estimate the cell fate trajectory due to the scarcity of cells in a transition state within a collected population of cells (Fig. 1d). This scarcity is caused by both the low frequency of cells entering a transition state and the short duration of the state itself^12–14^.

Another approach is a longitudinal analysis, such as live-cell imaging (LCI) techniques, that can reliably capture a transition state even if it is infrequent and short by visualizing the molecules associated with the fate determination process^2,9,12,15,16^. However, LCI analysis may not provide a comprehensive view of potential factors as it is limited to the predetermined molecular dynamics that are being visualized.

Here we report the development of the Time-Dependent Cell-State Selection (TDCSS) technique which combines the advantages of single-cell transcriptome analyses and LCI (Fig. 1e). TDCSS is a time-dependent cell recovery method that utilizes longitudinal cellular state information recorded using an LCI technique to identify the molecular signatures at the targeted transition state of the fate determination process (Fig. 1c, f), instead of relying on computational inference for time-dependent analysis. We demonstrate the effectiveness of the TDCSS technique by applying it to mouse group 2 innate lymphoid cells (mILC2s)^17,18^ that select their fate to induce a rapid type 2 immune response by producing a large amount of type 2 cytokines. We further demonstrate the power of the TDCSS technique by applying it to the rare human ILC2s (hILC2s)^19–21^ and obtaining the signature of expression in the transition state of the fate determination process.

## Results

### Development of the TDCSS technique

The key technical challenge of our method is integrating the destructive single-cell transcriptome analysis and the non-invasive LCI without compromising the benefits of either one (Fig. 1g). To overcome this challenge, we have designed and developed the TDCSS technique, which employs LCI to detect an index of the transition state, followed by the simultaneous recovery of indexed cells for subsequent transcriptome analysis. The TDCSS technique is composed of four steps: (1) Measurement of individual cell activity using LCI, (2) Real-time analysis of the activity trace, (3) Decisions on cell recovery, and (4) Recovery of the target cells. In the measurement step, images of individual cells are acquired using LCI. In the subsequent analysis step, the acquired images are analyzed in real-time to identify cells in the targeted activation state. In the third step, the activity of the identified cells is evaluated in a time-series manner to determine whether to flag them for recovery. Finally, in the recovery step, flagged cells are selectively harvested for transcriptome analysis.

### Evaluating the effectiveness of the TDCSS

As a proof of concept and to evaluate the effectiveness of the TDCSS technique, we selected the cytokine secretion response of mouse ILC2s (mILC2s) for the following reasons. First, mILC2s rapidly produce a large amount of type 2 cytokines, such as interleukin (IL)-5 and IL-13, during the activation process^17^, which makes the initiation of the transition state easily detectable by the live-cell imaging (LCI) technique. Second, mILC2s are activated by humoral factors, such as IL-33^17^, which facilitates the reconstitution of their activation response in microscopy experiments. Third, mILC2s are non-adherent cells, thus the flagged mILC2s can be easily recovered using a glass capillary.

**Fig. 2:**
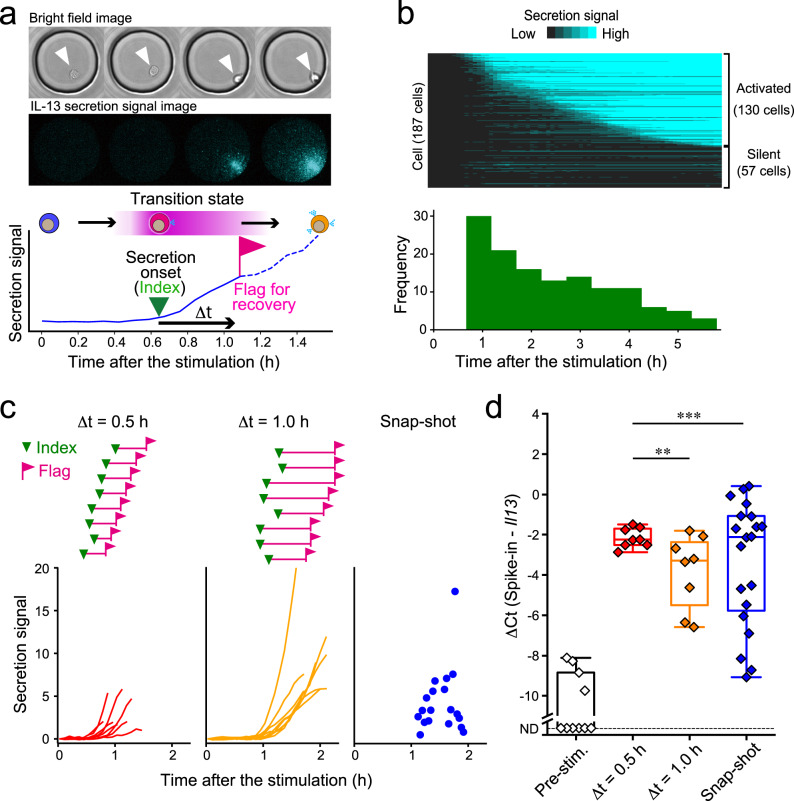
The TDCSS technique found a uniform *Il13* mRNA level of mILC2 in transition state. **a** Time-lapse images and intensity trace of IL-13 secretion signal from a stimulated mILC2 acquired by LCI-S. **b** Heatmap of IL-13 secretion signal from individual mILC2s and a histogram of their secretion onset times. **c** IL-13 secretion onset (inverted triangle), recovery timing (flag), and signal trace (bottom) of each mILC2 subjected to qRT-PCR analysis. **d** Box plots showing the median and interquartile range of *Il13* mRNA levels after stimulation. Whiskers represent minimum and maximum values. ND: not detected. **P < 0.01, ***P < 0.001, F-test.

To perform the TDCSS technique, we observed the IL-13 secretion activities of 187 individual ILC2s from mouse fat tissues stimulated by recombinant IL-33 using real-time single-cell imaging of protein secretion^12^ (LCI of secretion activity, LCI-S) (Fig. 2a). The traces of the secretion signal showed that cells started secretion after various latencies over several hours (Fig. 2b). We found that a delay time (⊿t) over 0.5 h from the onset of secretion (index) is needed to reliably flag the target cells (Supplementary Fig. 1).

To validate the TDCSS technique, we compared mRNA levels of *Il13*, which encodes IL-13, between “time-dependent” recovered cells using the TDCSS technique at ⊿t = 0.5 or 1.0 h (Supplementary Fig. 1) and “snap-shot” recovered cells only from secretion-positive mILC2s (Fig. 2c) using single-cell quantitative RT-PCR (qRT-PCR) (Fig. 2d, Supplementary Fig. 2). The variation of Il13 expression in mILC2s recovered in the “time-dependent” manner at ⊿t = 0.5 h was significantly reduced compared to that of mILC2s recovered in a “snap-shot” manner. Additionally, the variation in mILC2s at at ⊿t = 1.0 h was greater than the variation in mILC2s at ⊿t = 0.5 h. These results indicate homogeneity in the *Il13* mRNA level in the transition state of mILC2’s activation process among cells, which diversified within 1.0 h. In conclusion, we found that using the onset of IL-13 secretion as an index was effective in obtaining the intracellular molecular information of mILC2 in the transition state using the TDCSS technique.

### The transcriptome of mILC2 in the transition state

**Fig. 3:**
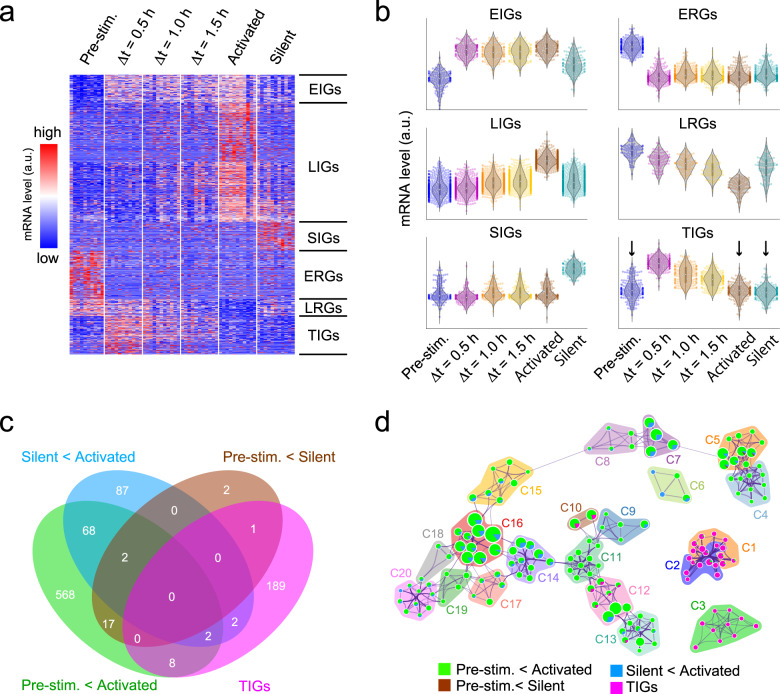
Unbiased characterisation of gene expression during ILC2’s activation process. **a** Heat map depicting the expression levels of 1471 differentially expressed genes (DEGs). **b** The trend of relative expression levels of genes in six transition classes. Additional information about each class can be found in Supplementary Data 1. **c** Overlap of the TIGs with the specified DEGs. **d** Network visualization of enriched ontology clusters of the TIGs and specified DEGs by using Metascape. Each node represents an enriched ontology cluster. The size of the node indicates the number of hit genes, while the pie chart illustrates the proportion of hit genes in the list. Clusters C1–C20, classified by similarity of enriched terms, are depicted in each coloured area. Additional information about each cluster can be found in Supplementary Data 2.

In order to explore the activation process of mILC2s comprehensively, we subjected mILC2s in the transition state (⊿t = 0.5, 1.0, and 1.5 h) and in other states (pre-stimulation: the state before the stimulation, activated: the state in which secretory activity was detected at 8 h of stimulation, when the majority of cells that had undergone the transition as shown in Fig. 2b, and silent: the state in which no secretion activity was detected at 8 h of stimulation) to single-cell RNA-seq (scRNA-seq). Based on the principal component analysis (PCA) or the trajectory inference (TI) results, we found that the transition cells were in a cluster/branch different from that of pre-stimulation cells or activated cells (Supplementary Fig. 3a, b). The TI method, which uses similarity between elements as an indicator, provides little analogy for abrupt changes in gene expression. Therefore, it is likely that the cells immediately after the change were displayed as the ends of different branches, due to the estimation of a pseudo-time different from the original time course (Supplementary Fig. 3b, c). To understand cell fate determination in rapid cell state transitions such as inflammatory responses, it is necessary to introduce real-time information obtained by our TDCSS method. To classify genes with variable expression during the activation process, we clustered differentially expressed genes (DEGs) between the activation states (one-way ANOVA *p* < 0.05). We classified 1,471 DEGs based on the transition modes in their expression levels into six classes: early induced genes (EIGs: 147), early reduced genes (ERGs: 254), late induced genes (LIGs: 627), late reduced genes (LRGs: 90), silent induced genes (SIGs: 151), and transiently induced genes (TIGs: 202) (Fig. 3a). Importantly, the TIGs could only be identified using the TDCSS technique because their expression levels were indistinguishable between the pre-stimulation and the activated / silent states (Fig. 3b, arrows). The difference in the dynamic nature of these transition modes explained why the pseudo-time estimated by the TI method differed from the actual elapsed time (Supplementary Fig. 3d). We further confirmed that the TIGs provided by the TDCSS technique were distinctive from the DEGs obtained by snapshot collection of pre- and post-stimulation cellular states (Fig. 3c). Interestingly, several genes reported to be involved in ILC2 activation were included in the TIGs we newly identified in TDCSS (Supplementary Data 1), for example, the growth inducers; *Rel* (cRel gene)^22^ and *Bcl2l1* (BCL-XL gene)^23^, the immune checkpoint factors; *Relb*^24^, *Klrg1*, *Lilrb4a* and *Cd274* (PD-L1 gene)^25^, the post-transcriptionally regulators; *Mir155hg* (host gene for miR-155)^26,27^ and *Zc3h12a* (Regnase-1 gene)^28^, the transcription factors; *Egr2*, *Nfkbiz* and *Nr4a1*^29^ and the anti-apoptotic protein; *Bcl3*^30^. The TIGs also contained *Il4* (type 2 cytokine IL-4 gene), *Il7rb* (IL-25 receptor gene), and *Tnfaip3* (A20 gene which is a negative regulator of IL-25 signals^31^). To study TIGs’ functions, we performed gene ontology (GO) analysis and found the relationships with immune activation-related terms such as “leukocyte activation” and “regulation of cytokine production” (Fig. 3d and Supplementary Data 2). The result that TIGs had a uniquely enriched ontology cluster indicates that the TDCSS technique succeeded in revealing the context of the transition state of mILC2’s activation process previously unavailable.

To investigate the influence of the index on the identification of TIGs, we reassigned the index to the onset of IL-5 secretion and re-evaluated the TIGs (Supplementary Fig. 4, Supplementary Data 3). Despite some shared GO terms between the IL-5 indexed TIGs and IL-13 indexed TIGs, these two groups of TIGs were found to be distinct (Supplementary Fig. 4e, f, Supplementary Data 4 and 5). We explored the relationship between the initiation of IL-13 and IL-5 secretion from individual cells during the activation process using two-colour LCI-S (Supplementary Fig. 5). The results indicate that under the experimental conditions described in this study, most mILC2s initiate secretion of both IL-5 and IL-13 simultaneously or IL-13 first. This suggests that the index based on IL-13 is able to detect an earlier transition state in the activation process. These findings demonstrate that the TDCSS technique can be employed to comprehensively characterize the gene expression in the transition state of the mILC2 activation process.

### Identification of TIGs of human ILC2

**Fig. 4:**
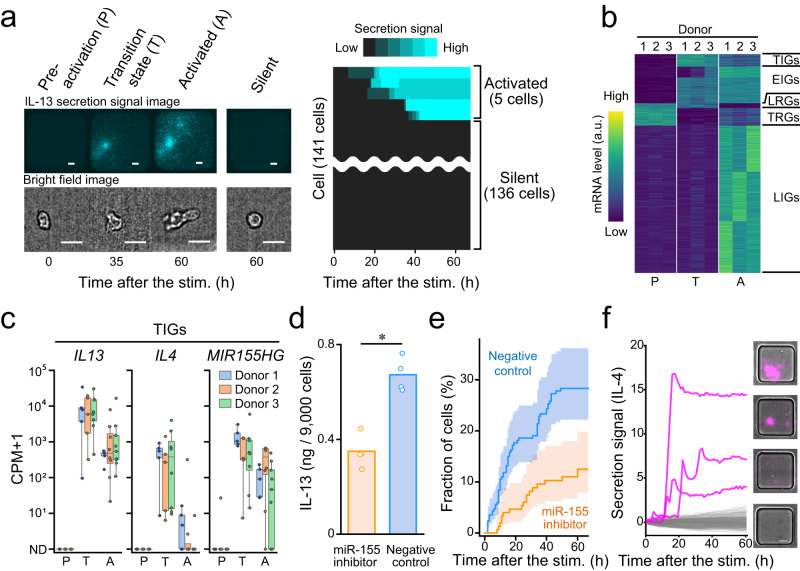
Identification of genes expressed in different activation steps from human specimens. **a** Representative time-lapse images of secretion signal and cell morphology (left) and heatmap of IL-13 secretion signal (right) of individual hILC2s. Scale bar, 25 µm. **b** Heatmap showing the average mRNA levels in each activation state of each specimen in standard scores (z-score). Details of each class are provided in Supplementary Data 6. **c** Box plot illustrating the expression levels of representative TIGs; *IL13*, *IL4* and *MIR155HG* in each activation state. Each colour represents a different specimen. The centre line, box limits, whiskers, and dots indicate the median, upper and lower quartiles, 1.5x interquartile range and individual cells, respectively. ND: not detected. **d** Comparison of IL-13 production in cultured human ILC2 with and without miR-155 inhibitor by ELISA of bulk culture supernatants. Inhibition was performed by electroporation of a synthetic miRNA inhibitor with miR-155 specific or control sequence. **e** Comparison of temporal changes in the estimated proportion of IL-13-secreting cells in cultured human ILC2s with and without miR-155 inhibitor using the Nelson-Aalen estimator with their respective 95% confidence intervals (coloured bands). The number of cells in negative control and inhibition were 259 and 153, respectively. The difference between them was compared with the log-rank test (observed *p* value = 0.0013). **f** Secretion signal traces and images of IL-4 producing individual hILC2s. Three secretion-positive traces are shown in magenta and other traces are shown in grey (388 traces).

Given the demonstration of the efficacy of the TDCSS technique through experiments utilizing expanded culture mILC2s, we subsequently analysed the expression signature of human ILC2s (hILC2s)^20,21,32^ during the transition state. Besides being a rare cell type in peripheral blood (50–150/mL)^20^, we confirmed that the population of hILC2s that initiated secretion (2–4%) exhibited a wide range of latency within 60 h after stimulation (Fig. 4a, Supplementary Fig. 6a, b), as previously reported (Supplementary Fig. 6c)^33^. Given the populational rarity and temporal heterogeneity, it is challenging to efficiently collect cells in the transition state of activation using a “snapshot” approach (Supplementary Fig. 6b).

We applied the TDCSS technique to hILC2 by indexing the onset of IL-13 secretion and recovered the hILC2s in the transition state (⊿t within 3 h, Supplementary Fig. 6d). We compared the transcriptome among hILC2s of three donors in the transition state and other states (pre-stimulation and activated at 60 h of stimulation or later). As a result, we identified 91 TIGs as well as genes in four other classes of transition patterns (EIGs:270, LIGs:1081, LEGs:35 and transiently reduced genes: TRGs 128) from 1605 DEGs (ANOVA *p* < 0.01) (Fig. 4b and Supplementary Data 6). Another approach was to identify TIGs for genes whose expression levels transiently increase and subsequently decrease, and the robustness of five of these TIGs (*IL17RB*, *CYTIP*, *IL13*, *IL4*, *MIR155HG*) was confirmed (Supplementary Fig. 7). As with the results in mILC2s, the dynamic nature of the transition patterns, especially TIGs, explained why the pseudo-time estimated by the TI method differed from the actual elapsed time (Supplementary Fig. 8).

Next, to confirm the functional involvement of TIGs in the activation process of ILC2, we investigated the roles of *IL4* and *MIR155HG* (Fig. 4c). Using LCI-S, we verified the contribution of miR-155 to ILC2 activation and found that the inhibition of miR-155 attenuated the stimulus-dependent activation of expanded culture hILC2s (Fig. 4d, e). IL-4 is a key inducer of type 2 immune response^34^ and plays an important role in hILC2 function, including proliferation and maintenance of the CRTH2 expression^35^. Although hILC2s have been reported to produce IL-4^19^, it was difficult to characterize the IL-4 secretion dynamics of hILC2s due to their low production of IL-4. Therefore, we examined IL-4 secretion dynamics from individual hILC2s by LCI-S and detected their transient secretion for several hours (Fig. 4f). Although we could propose the plausibility of transient IL-4 production, further exploration of experimental conditions is required to reproduce the protein-level secretion of IL-4.

## Discussion

We succeeded in a comprehensive exploration of the potential regulators of fate determination by developing and utilizing the TDCSS technique to recover individual cells at the transition state, even though such potential regulators existed in a small population of cells and only for a short duration. The discovery of a uniquely enriched gene ontology cluster of the transiently induced genes (Fig. 3d) indicates that the TDCSS technique is able to exploit the IL-13 secretion onset to identify the characteristic state during the activation process of ILC2.

The process by which activated ILC2s determine their fate to produce a tremendous amount of cytokine^17,36^ was previously unknown. *IL4* and *MIR155HG*, whose respective products IL-4 and miR-155 are two positive regulators of the cytokine production and proliferation of ILC2^26,35^, are upregulated in the transition state of the activation process, suggesting that they are key regulators of the subsequent activation process. MiR-155 is known to target and repress IL-33 signalling repressors, including *INPP5D*^36–38^ and *DUSP10*^39,40^. Although these genes were not expressed immediately after the secretion onset of IL-13, they were expressed at the late stage of the activation process under IL-33 stimulation (Supplementary Fig. 7c). *MIR155HG* may be prepared to silence these negative regulators from the transition state of the activation process to maintain ILC2s’ proliferation and their cytokine production activity. Furthermore, miR-155 also targets *SOCS1*^41,42^ and *DUSP4*^43,44^, both encoding inhibitors of IL-2 signalling that support ILC2’s activation^45,46^, and *IFNGR1*^47^, encoding a receptor of IFN-γ that suppresses ILC2’s activation^48^. This indicates that miR-155 contributes to the fate determination in several ways, however, more detailed observations are required to understand whether miR-155 regulates by a transcriptional or a translational repression. IL-4^35^, the other positive regulator we focused on, was confirmed to be transiently produced at the protein level. Our data show a constant expression of *IL4R*, which encodes an IL-4 receptor. This suggests that IL-4 produced by ILC2 early in its activation process may contribute to the positive regulation of activation in an autocrine manner, although further research is needed to confirm this hypothesis. The genes expressed in the transition state of the activation process that currently have an unknown function in the ILC2 should be studied in the future in order to gain a deeper understanding of the mechanism of fate determination in ILC2.

The selection of surface antigens for human ILC2 (hILC2) remains a topic of ongoing research, and the TDCSS technique has the potential to provide new insights not only in identifying transiently induced genes, but also in selecting surface antigens that index the activation of hILC2. Liu et al. have reported that *CD30*, *TNFR2*, and *ICOS* are newly proposed surface antigens that define ILCs producing type 2 cytokines^49^. In contrast, our TDCSS results revealed that *CD30*, *TNFR2*, and *ICOS* were stably expressed on ILC2 after activation, while *CRTH2 (PTGDR2*) and *IL7R* were downregulated on ILC2 after activation (Supplementary Fig. 9). These data suggest that the newly proposed surface antigen markers identified by Liu et al. are markers of activated ILC2. This activation-dependent change in surface antigens is likely to be observed not only in ILC2, but also in various immune cells, and thus the TDCSS technique will greatly contribute to the identification of surface antigen markers that reflect not only the cell type, but also the stage of activation.

While we have succeeded in exploring the activation process in ILC2, the TDCSS technique has yet to reveal the full picture of the fate-determination mechanism. Towards this future goal, TDCSS still faces a number of technical challenges. Firstly, TDCSS must increase its current throughput, which is currently hindered by the time-consuming manual recovery steps and scanning of cellular images. Secondly, the information obtained by TDCSS is dependent on the choice of index used to define the transition state. In this study, different TIGs were extracted when IL-13 and IL-5 were used as indexes (Supplementary Fig. 4), indicating that different indexes capture different aspects of the transition state. Therefore, it will be necessary to use multimodal information based on LCI, such as cell morphology, motility, and secretion, to achieve a comprehensive understanding of the fate-determination process.

While techniques exist to record the transcriptional histories of cells^50–52^, it is difficult to analyse rare clinical specimens as TDCSS has achieved, due to the need for genetic modification or aggregation of information from a large number of cells. While we have focused on the activation process of ILC2 in this study, many LCI techniques have been developed to trace various fate determination processes, including immune response^15^, cell differentiation^53,54^, cell death^2^, and stimulus response^9,55^. By utilizing these LCI techniques, the TDCSS technique can aid in the elucidation of the context of these fate determination processes. Additionally, it is known that in some immune responses, secretion is initiated immediately after the initiation of the transition state, *via* post-transcriptional or post-translational regulation^56–59^. Using an index that does not include delays due to transcription, allows for exploration of earlier stages of the fate-determining process.

## Methods

### Reagents

For cytokine secretion imaging using the LCI-S, we used anti-human IL-13 (MAB213 and BAF 213) and IL-4 (MAB604 and BAF204), anti-mouse IL-13 (MAB413 and BAF413) and IL-5 (MAB405 and BAM705) antibodies (R&D Systems, Minneapolis, MN, USA). For the measurement of IL-13 in culture supernatant, we used enzyme-linked immuno-sorbent assay (ELISA) kit (Human IL-13 Quantikine ELISA Kit, R&D Systems). For cell sorting of hILC2s, we used lineage antibody cocktails of human CD3 (UCHT1), CD14 (HCD14), CD16 (3G8), CD19 (HIB19), CD20 (2H7), CD56 (HCD56), PE-conjugated anti-human CD127 (A019D5), PE/Cy7-conjugated anti-human CD161 (HP-3G10) antibodies (BioLegend, San Diego, CA, USA), and Alexa Fluor 647-conjugated anti-human CRTH2 (BM16) antibody (BD Biosciences, San Jose, CA, USA).

For hILC2 stimulation, we used recombinant human IL-2 (Imunace35, Shionogi & Co., Ltd, Japan), IL-33, and TSLP (3625-IL/CF and 1398-TS/CF, R&D Systems, Minneapolis, MN, USA). For mILC2 stimulation, we used recombinant mouse IL-2 and IL-33 (402-ML and 3626-IL, R&D Systems).

For the miR-155 functional assay for hILC2 activation, we used miR-155 inhibitor (MISSION synthetic miRNA inhibitor, Sigma-Aldrich) and control (MISSION synthetic miRNA inhibitor negative control #1).

### Isolation of hILC2s

Peripheral blood was obtained from three volunteers at Keio University School of Medicine. The study protocol was approved by the Institutional Review Board (IRB) of the Keio University School of Medicine (IRB approval number: 20090009). All subjects provided their written informed consent. For isolation of ILC2s from human peripheral blood, mononuclear cells were obtained using Lymphoprep^TM^ (Axis-Shield, Dundee, UK), according to the manufacturer’s protocols. Lineage-negative (CD3, CD14, CD16, CD19, CD20 and CD56) and CD45-, CD127-, CRTH2- and CD161-positive cells were sorted using a MoFlo^TM^ XDP flow cytometer (Beckman Coulter, Brea, CA, USA).

### Preparation of mILC2

We isolated mILC2s from mouse fat tissues using a previously described protocol^60^. Briefly, we obtained pooled mesentery from 20 wildtype C57BL/6 mice. Lineage-negative cells were selected by negative selection using Microbeads and AutoMACS (Miltenyi Biotec GmbH). Then, c-Kit+Sca-1+ cells were sorted as ILC2s using a FACSAria flow cytometer (BD Bioscience). The acquired mILC2s were cultured for several months in RPMI-1640 media (R8758, Sigma-Aldrich, St. Louis, MO, USA) containing 10% foetal calf serum (FCS) (S1820, Japan Bioserum, Hiroshima, Japan), 10 mM HEPES buffer (H3537, Sigma-Aldrich), 1 mM sodium pyruvate (11360-070, Thermo Fisher Scientific, Waltham, MA, USA), 1x non-essential amino acids (M7145, Sigma-Aldrich), 100 U/mL penicillin and 100 µg/mL streptomycin (15140-122, Thermo Fisher Scientific), 14.3 mM 2-mercaptoethanol (21985023, Thermo Fisher Scientific), and 10 ng/mL recombinant mouse IL-2 (1399-IL, R&D Systems) in 96-well round-bottom plates. ILC2 cells were usually isolated from retired female breeder mice unless stated otherwise. All experiments were approved by the Animal Care and Use Committee of RIKEN or Keio University and performed according to institutional guidelines.

### LCI of secretion activity of ILC2s

A fully automated inverted microscope (ECLIPSE Ti-E; Nikon, Tokyo, Japan) was used for time-lapse imaging using total internal reflection fluorescence (TIRF) illumination of LED light (X-Cite XLED1, mounted with RLX: 615 -655 nm, Excelitas Technologies Corp., Waltham, MA) through white-light TIRF optics (high-performance Epi-fl illuminator module TI-SFL). The optical configurations used were the following: excitation filter = FF01-635/18; emission filter = FF01-692/40; and dichroic mirror = FF560/659-Di01. These optical filters were purchased from Semrock (Rochester, NY, USA). We introduced pre-stimulation ILC2s into a microfabricated well array dish, which composed with a polydimethylsiloxane (PDMS), amorphous fluorocarbon polymer CYTOP^TM^, and glass with the immobilised capture antibody. We selected wells that contained single cells and immediately started scanning after replacing the medium with detection medium containing CF660R-labelled detection antibody and cytokine stimuli.

In the hILC2s experiment, we used a detection medium containing 10 U/mL IL-2, 25 ng/mL IL-33, and 25 ng/mL TSLP. For mILC2s, we used detection medium containing 10 ng/mL IL-2 and 10 ng/mL IL-33. Scan intervals were 15 min for hILC2s experiments and 4 min for mILC2s experiments.

When the secretion dynamics of the two types of cytokines were measured separately, a mixture of IL-13 and IL-5 capture antibodies was fixed on the bottom of the chip, and IL-13 and IL-5 detection antibodies are labelled with fluorescent substances of different wavelengths (CF660R or Cy3). Then, we perform live-cell imaging by switching between two sets of filters and lighthouse of different wavelengths to obtain separate signal increases at each wavelength.

### Functional assay using miR-155 inhibitor

We obtained hILC2 from peripheral blood, proliferated by culturing in the presence of IL-2 and IL-33, followed by 5 days of culture with IL-2 alone. Electroporation was performed using Neon Electroporation System (Thermo Fisher Scientific) at 1600 V, 10 ms, 3 pulses in T buffer. miR-155 inhibitor or control was introduced at a final concentration of 0.1 uM. After electroporation, the cells were incubated overnight and introduced into the LCI-S platform to measure the secretion of IL-13 under the same conditions as in other experiments. In the bulk experiment, 9000 cells were incubated with IL-2 (10 U/mL), IL-33 (25 ng/mL), and TSLP (25 ng/mL) for 2 days, following which the IL-13 concentration in the supernatant was measured by ELISA.

### Detection of the secretion onset

For detection of increased secretion signals from each cell, acquired images were sequentially analysed using Python 3 software developed in-house (https://github.com/TanaYumi/TDCSS). The program calculated the mean intensity of each well and compared the intensity to the first image intensity. The resulting value was used as the secretion signal. When the last three secretion signals were higher than the value estimated using linear regression of the past signal by one standard deviation, the program notified the operator of potential secretion onset.

After the experiments, we reanalysed the secretion signals to determine the accurate time between secretion onset and cell recovery (⊿t). The secretion onset time was determined by fitting the values to the following formula (1):

1 $${signal}\,\left(t\right)=\left\{\begin{array}{c}{at},\hfill {{{{{\rm{\& }}}}}}0\, < \, t \, < \, \hat{t}\\ b\left(t-\hat{t}\right)+a\hat{t},{{{{{\rm{\& }}}}}}t\ge \hat{t}\end{array}\right.$$

In the formula (1), $a$ represents drift, $b$ represents the slope of the secretion signal increase, $t$ represents the time after stimulation and $\hat{t}$ represents the time of secretion onset. The initial value of $\hat{t}$ was set to the visually determined onset on the automated image-analysis system. ⊿t was obtained by calculating the interval between secretion onset and cell recovery.

### Cell recovery

Single cells or single colonies were recovered using a glass capillary (L-Tip 15 µm 60° 15 mm, Yodaka Co., Ltd., Kawasaki, Japan) and a pneumatic microinjector (IM-11-2, NARISHIGE, Tokyo, Japan). The glass capillary was positioned using a micromanipulator (TransferMan 4r, Eppendorf, Hamburg, Germany, or Quick Pro, Micro Support Co. Ltd., Shizuoka, Japan). The aspirated cell was ejected to 2.5 μL RNase-free water (06442-95, Nacalai tesque, Kyoto, Japan) for RNA-seq or 2.5 μL of the qRT-PCR reaction mixture (CellsDirect One-Step qRT-PCR Kits, Thermo Fisher Scientific) for qRT-PCR in a PCR tube.

The recovered cells were immediately frozen in liquid nitrogen and stored at -80°C for subsequent gene expression analysis. The pre-stimulation cells were recovered prior to the addition of the stimulus from the LCI-S platform. In the experiments involving mILC2s, activated and silent cells were randomly recovered from cells displaying positive and negative secretion signals 8 h after the initiation of stimulation. In the experiments involving hILC2s, activated and silent cells were randomly recovered 36 or 60 h (from 2 specimens) and 120 h (from 1 specimen) after the initiation of stimulation.

### qRT-PCR

To increase the accuracy of single-cell mRNA quantification, we added the same amount of ERCC Spike-In RNA (622,819 copies per cell) to each sample and used it as a reference. cDNA was synthesised using CellsDirect One-Step qRT-PCR Kits (Thermo Fisher Scientific) using TaqMan probes targeting four genes (*Il13*, *Il5*, *Gapdh* and *Rplp0*) and four spike-in RNA sequences (ID130, ID136, ID131 and ID85). The TaqMan probes used were: *Il13* (Mm.PT.58.31366752), *Il5* (Mm.PT.58.41498972), *Gapdh* (Mm_GAPDH), *Rplp0* (Mm.PT.58.43894205) purchased from IDT Integrated DNA Technologies (Coralville, IA, USA), ID130 (Ac03459943_a1), ID136 (Ac03459946_a1), ID131 (Ac03459944_a1) and ID85(Ac03459923_a1) purchased from Thermo Fisher Scientific. The PCR product was diluted ten-fold in RNase-free water, and 1 μL of the PCR product was analysed using qPCR using TaqMan Fast Universal PCR Master Mix (Thermo Fisher Scientific). Relative gene expression was calculated using the ΔΔCt method using ID136 or *Gapdh* as a reference. Samples, where *Gapdh* or *Rplp0* could not be detected, were omitted from the analysis.

### RNA sequencing

We synthesised cDNA libraries using SMART-Seq v4 3’-DE Kits (Takara Bio Inc., Shiga, Japan) according to the manufacturer’s instructions, with some modifications, adding the synthetic oligo RNA/DNA to suppress undesirable concatemers. A total of 3,892 copies of ERCC spike-in RNA were added to each sample. The cDNA was purified using Agencourt AMPure XP magnetic beads (Beckman Coulter). Library quality check was performed using an Agilent 2100 Bioanalyzer (Agilent Technologies, Santa Clara, CA, USA) and Agilent High Sensitivity DNA Kits (5067-4626). Degraded or low-yield samples were removed. Qubit High Sensitivity assays (Thermo Fisher Scientific) were performed to quantify the cDNA in each library, and 5 or 6 libraries with different indexed primers were evenly pooled. Pooled cDNA (400 pg) was tagmented using Nextera XT DNA Library Prep Kits (Illumina, San Diego, CA, USA), as described in the protocol. Library size and cDNA amount were quantified using an Agilent High Sensitivity DNA Kit and Qubit High Sensitivity assays, respectively. Pooled libraries were sequenced using 91-bp paired-end sequencing on a MiSeq instrument (Illumina). After library demultiplexing and adaptor trimming, we aligned read 1 to reference sequences (human: GRCh38.90, mouse: GRCm38) using TopHat^61^, and the read counts were calculated using HTSeq^62^.

In mILC2 experiment, total 103 transition cells were collected using TDCSS. We reanalysed the secretory signals of these cells, determined the exact ⊿t, and classified them into cells with ⊿t = 0.5 h, 1.0 h, and 1.5 h. The collected cells were sequentially prepared into cDNA libraries, and library preparation was repeated until 11 libraries of each Δt were obtained. As a result, we obtained 33 cDNA libraries (11 libraries for each ⊿t = 0.5 h, 1.0 h, and 1.5 h), all passed QC at the post sequencing analysis. In hILC2 experiment, 13, 9, 13 transition cells were collected using TDCSS from each donor and library preparation was conducted for all cells, resulting in 5, 5, 8 transition cells passed QC at the post sequencing analysis.

### Principal component analysis

PCA was performed using the Python 3 module “Scikit-learn^63^”. All cells’ expression data were used for the analyses. Count data of all genes were normalized by count per million (CPM). We used all genes for the PCA, except for genes with log2(CPM + 1) > 1 under 9 cells.

### Pseudo-time analyses

The pseudo-time analysis was performed using Monocle^64^(2.20.0). All cells’ expression data were used for the analyses. Genes with an average expression level of less than 1 across cells were removed from further analysis. After that, highly variable genes were obtained by PCA and used for the trajectory inference. Used software versions and parameters of each processes can be found in the scripts at (https://github.com/TanaYumi/TDCSS).

### Enrichment analysis

We used Metascape^65^ for enrichment analysis using default settings.

### Statistics and reproducibility

Variety of the expression levels acquired by qRT-PCR experiment was tested by F-test.

To identify the differentially expressed genes among activation cell states (pre stimulation, transition state, activated and Silent for mouse ILC2s), one-way ANOVA was performed. In the analysis of mILC2, the earliest transition state (Δt = 0.5 h) was used for comparison. In the analysis of hILC2, we used expression data from three donors. The expression average among the same states in individuals were used for the clustering to avoid the bias caused by the difference in the number of cells among the states. Genes with *P* < 0.05 (mILC2) or < 0.01 (hILC2) were classified as highly variable. We used these highly variable genes for subsequent analyses.

Differential expression analysis of activated > pre-stimulation, silent > pre-stimulation and activated > silent states were performed using the Mann-Whitney rank test by SciPy 1.0^66^ on genes that were expressed in more than four cells and had a fold-change 4 or above. Genes with an adjusted *p* value < 0.05 (adjusted using the Benjamini-Hochberg method with FDR < 0.1) were selected as differentially expressed genes.

### K-Means clustering

K-Means clustering was performed with the Python 3 module “Scikit-learn^63^”. First, we classified the genes into 14 classes for mILC2 and 8 classes for hILC2 by the K-Means method. We grouped clusters with similar variation patterns into the same class, resulting in six or five classes, respectively.

### Reporting summary

Further information on research design is available in the Nature Portfolio Reporting Summary linked to this article.

### Supplementary information

Supporting Information

Description of Additional Supplementary Files

Supplementary Data 1

Supplementary Data 2

Supplementary Data 3

Supplementary Data 4

Supplementary Data 5

Supplementary Data 6

Supplementary Data 7

Reporting Summary

## Data Availability

RNA-sequencing data will be available through the GEO database; mILC2 expression data (GSE222762), hILC2 expression data (GSE213810, GSE222761). Source data for main figures are provided with the supplementary data 7. Other datasets generated during and/or analysed during the current study are available from the corresponding authors on reasonable request.

## Code availability

Code is available at: https://github.com/TanaYumi/TDCSS. Environments for gene expression analysis are available at: https://hub.docker.com/r/yumikotanaka/tdcss.
