# Supplementary material for: Time-dependent cell-state selection identifies transiently expressed genes regulating ILC2 activation
Source: Commun Biol. 2023 Sep 6;6:915. doi: 10.1038/s42003-023-05297-w (PMC10482971; doi:10.1038/s42003-023-05297-w)
Supplement: Supplementary file 1 — Supporting Information [file 42003_2023_5297_MOESM1_ESM.docx]

**Supporting Information:**

**Time-Dependent Cell-State Selection identifies transiently expressed genes regulating ILC2 activation**

Yumiko Tanaka^1^, Mai Yamagishi^2,3^, Yasutaka Motomura^4^, Takashi Kamatani^1,5,6,7^, Yusuke Oguchi^8,9^, Nobutake Suzuki^2^, Tsuyoshi Kiniwa^10^, Hiroki Kabata^7^, Misato Irie^7^, Tatsuhiko Tsunoda^1,10,11^, Fuyuki Miya^12^, Keisuke Goda^13,14,15^, Osamu Ohara^16^, Takashi Funatsu^2^, Koichi Fukunaga^7,17^, Kazuyo Moro^4,10,17^, Sotaro Uemura^1,17*^ and Yoshitaka Shirasaki^2,17*^

^1^ Department of Biological Sciences, Graduate School of Science, The University of Tokyo, Tokyo, Japan

^2^ Graduate School of Pharmaceutical Sciences, The University of Tokyo, Tokyo, Japan

^3^ Live Cell Diagnosis, Ltd., Saitama, Japan

^4^ Department of Microbiology and Immunology, Graduate School of Medicine, Osaka University, Osaka, Japan

^5^ Department of AI Technology Development, M&D Data Science Center, Tokyo Medical and Dental University, Tokyo, Japan

^6^ Division of Precision Cancer Medicine, Tokyo Medical and Dental University Hospital, Tokyo, Japan

^7^ Division of Pulmonary Medicine, Department of Medicine, Keio University School of Medicine, Tokyo, Japan

^8^ PRESTO, JST, Saitama, Japan

^9^ RIKEN Cluster for Pioneering Research, Saitama, Japan

^10^ RIKEN Center for Integrative Medical Sciences, Kanagawa, Japan

^11^ Department of Computational Biology and Medical Sciences, Graduate School of Frontier Sciences, The University of Tokyo, Tokyo, Japan

^12^ Center for Medical Genetics, Keio University School of Medicine, Tokyo, Japan

^13^ Department of Chemistry, Graduate School of Science, The University of Tokyo, Tokyo, Japan

^14^ Department of Bioengineering, University of California, Los Angeles California, USA

^15^ Institute of Technological Sciences, Wuhan University, Hubei, China

^16^ Kazusa DNA Research Institute, Chiba, Japan

^17^ These authors jointly supervised this work

* To whom correspondence should be addressed: [shirasaki@g.ecc.u-tokyo.ac.jp](mailto:shirasaki@g.ecc.u-tokyo.ac.jp) or [uemura@bs.s.u-tokyo.ac.jp](mailto:uemura@bs.s.u-tokyo.ac.jp)


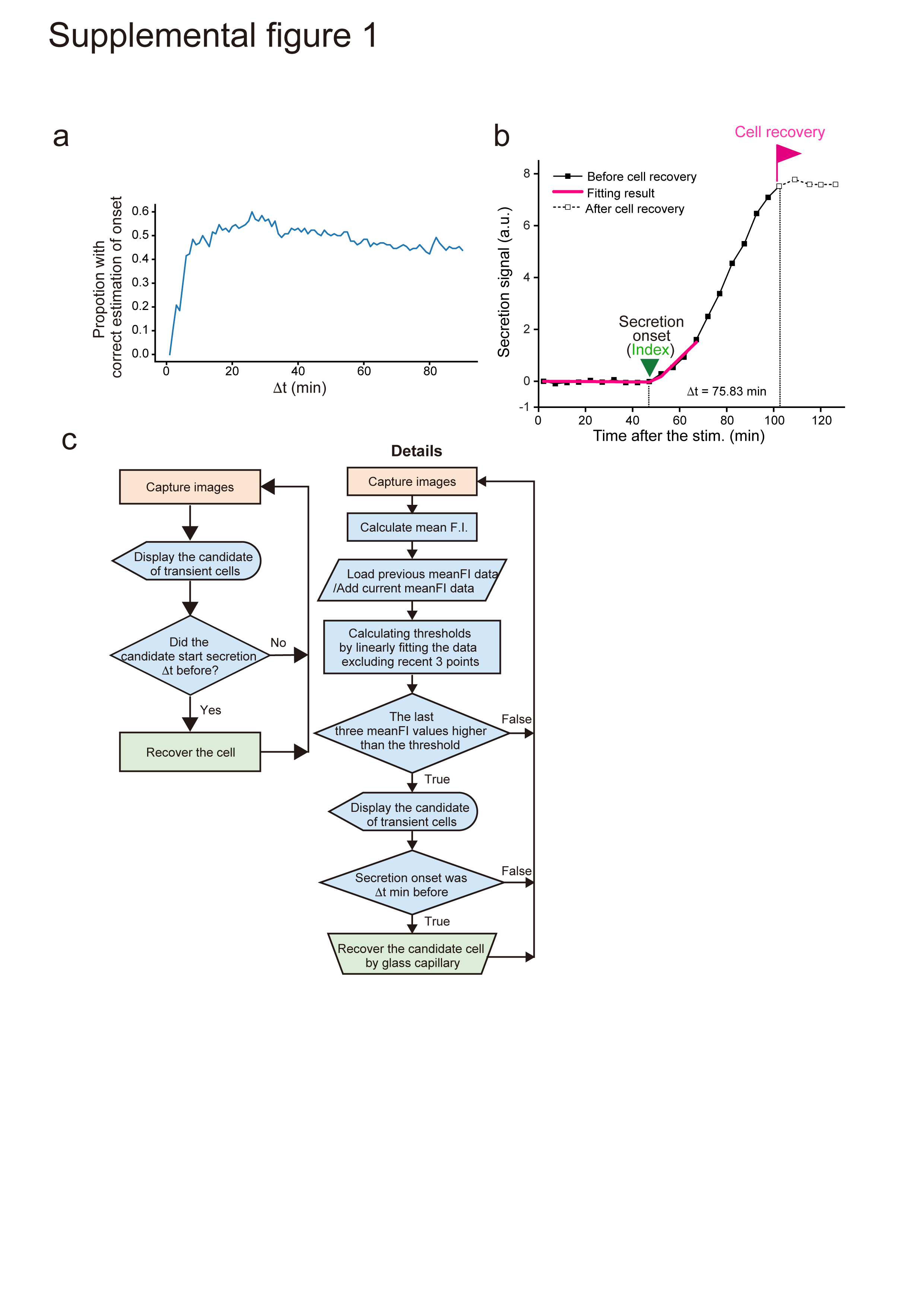


**Supplementary Fig. 1| Detection of Secretion Onset.**

(a) The trade-off between the time from secretion onset to recovery flag (Δt) and the accuracy of index determination is depicted. The vertical axis illustrates the proportion of traces whose standard deviation is lower than 10 minutes, when the secretion onset is determined by fitting traces from the start of measurement to Δt minutes after the secretion onset (as outlined in Supplementary Figure 1c and the Methods section). (b) The methodology used to determine the timing of secretion onset through post-analysis is illustrated. The secretion signal trace is fitted to an elbow-shaped function (as outlined in the Methods section). (c) A simplified (left) and detailed (right) flowchart depicting the operation of the TDCSS technique is presented. Red, blue, and green correspond to the operations of measurement, analysis, and recovery, respectively.

**
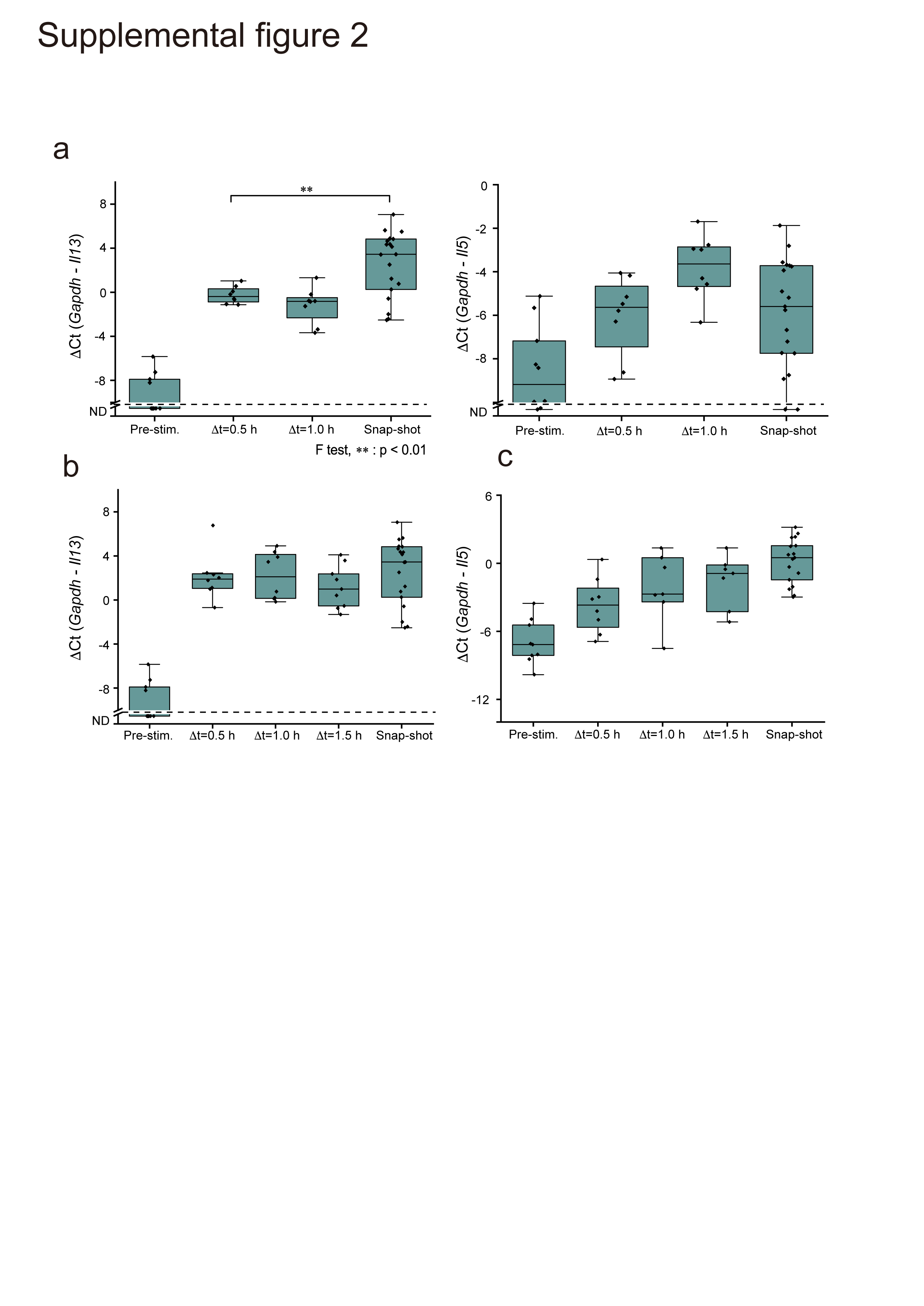
Supplementary Fig. 2| qRT-PCR Results of Single-mILC2s Recovered by IL-13 Index or IL-5 Index**(a) *Il13* (left) and *Il5* (right) Gapdh-corrected expression levels of cells shown in Figure 2d, which illustrates ERCC-corrected *Il13* expression levels. (b) *Il13* expression levels of cells recovered using IL-13 as an index. (c) *Il5* expression levels of cells recovered using IL-5 as an index. The pre-stimulation and Δt = random cells shown in a, b, and c are the same cells as those in Figure 2d.

**
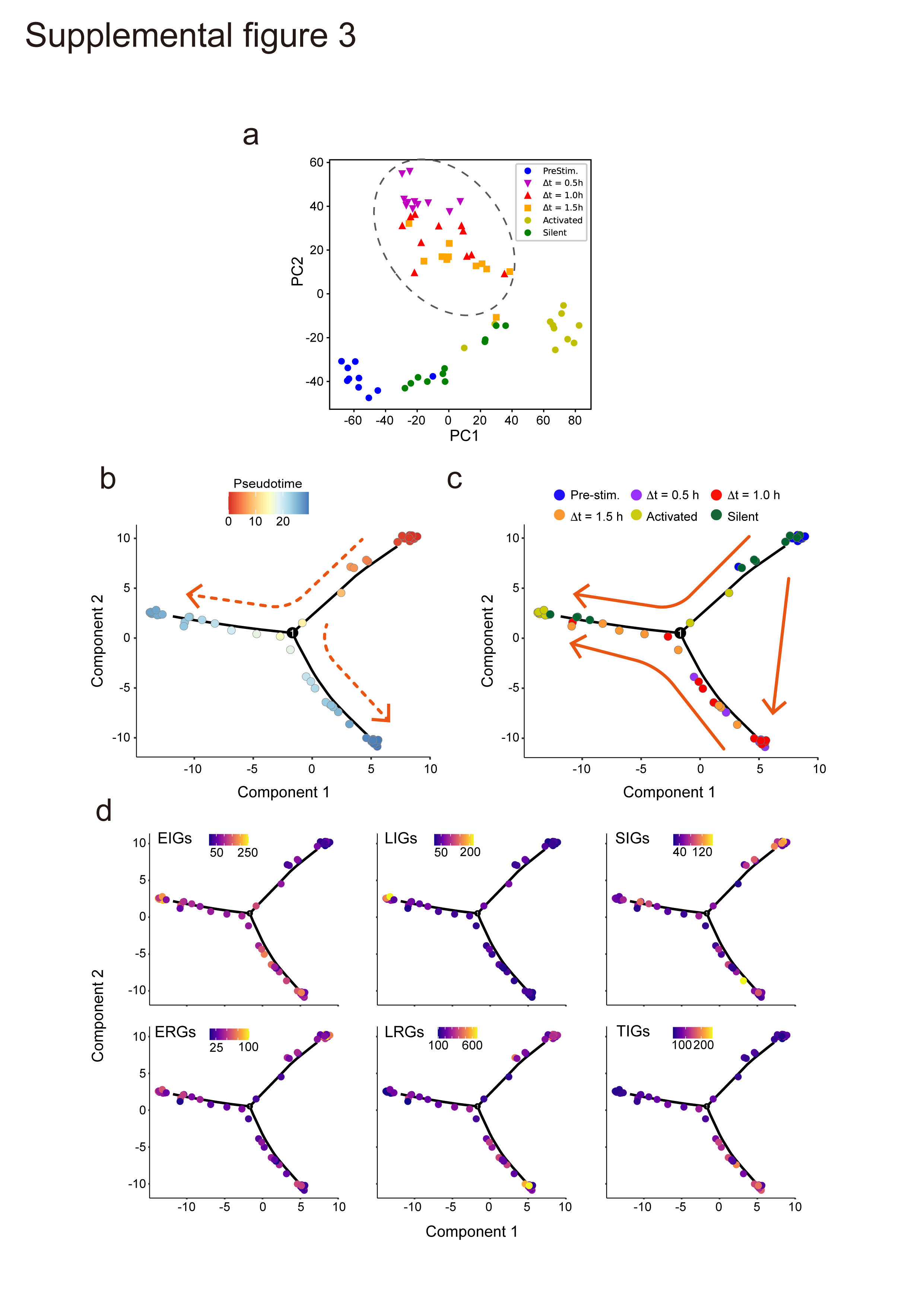
**

**Supplementary Fig. 3| Pseudotemporal Analysis of mILC2 Activation Process**

(a) Principal component analysis of gene expression in cells recovered using IL-13 as an indicator, with the area of transition cells clustered circled by a dashed line. (b-d) Results of trajectory inference by Monocle, with (b) pseudotime depicted in color, (c) activation states indexed by the TDCSS technique depicted in color, and (d) average expression levels of gene groups classified into six expression transition patterns by the TDCSS technique depicted in color. The dashed arrows and solid arrows indicate the flow of state transitions as indicated by pseudotime and the TDCSS technique, respectively. Notably, LIGs and TIGs were highly expressed at the respective node ends after branching.

**
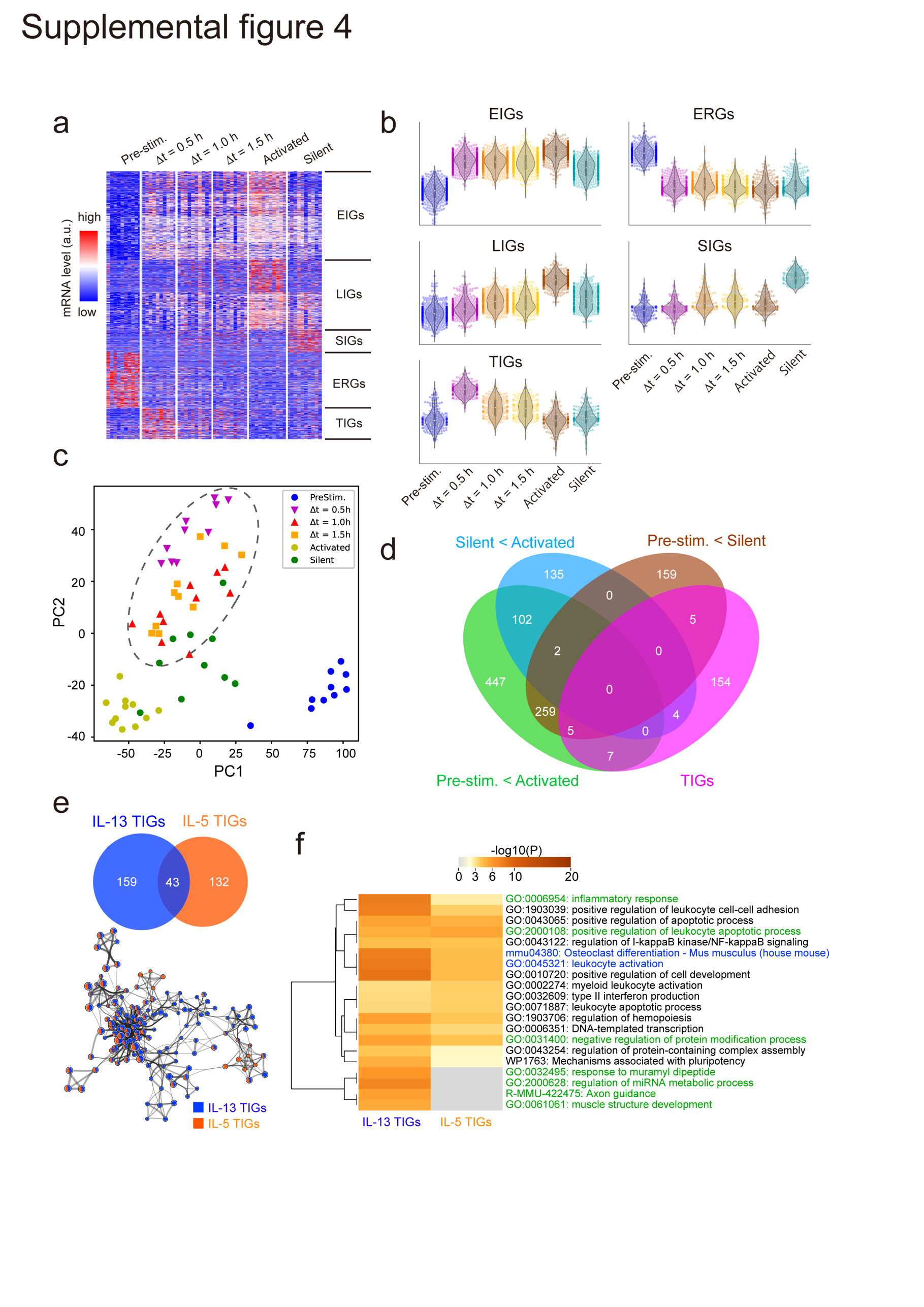
**

**Supplementary fig. 4| TDCSS analysis of mILC2 using IL-5 as an index**

(a) Heatmap depicting the relative expression levels of 1,438 differentially expressed genes in cells at various stages of activation. The horizontal axis indicates the stages of activation, and the vertical axis displays each gene, grouped according to their expression transition class (EIGs: early induced genes, LIGs: late induced genes, SIGs: silent induced genes, ERGs: early reduced genes, TIGs: transiently induced genes). (b) The trend of relative expression levels of genes in five transition classes. (c) Principal component analysis of gene expression in cells recovered using IL-13 as an indicator. (e) Venn diagram illustrating the overlap of TIGs obtained using IL-13 or IL-5 as an index and the respective enrichment ontology clusters by Metascape. (f) Heatmap of accumulative hypergeometric p-values of each enrichment term. The terms that were particularly enriched in the IL-13 TIG in Figure 3d are highlighted in blue (matched with GO in top 20) and green (GO in top 100).


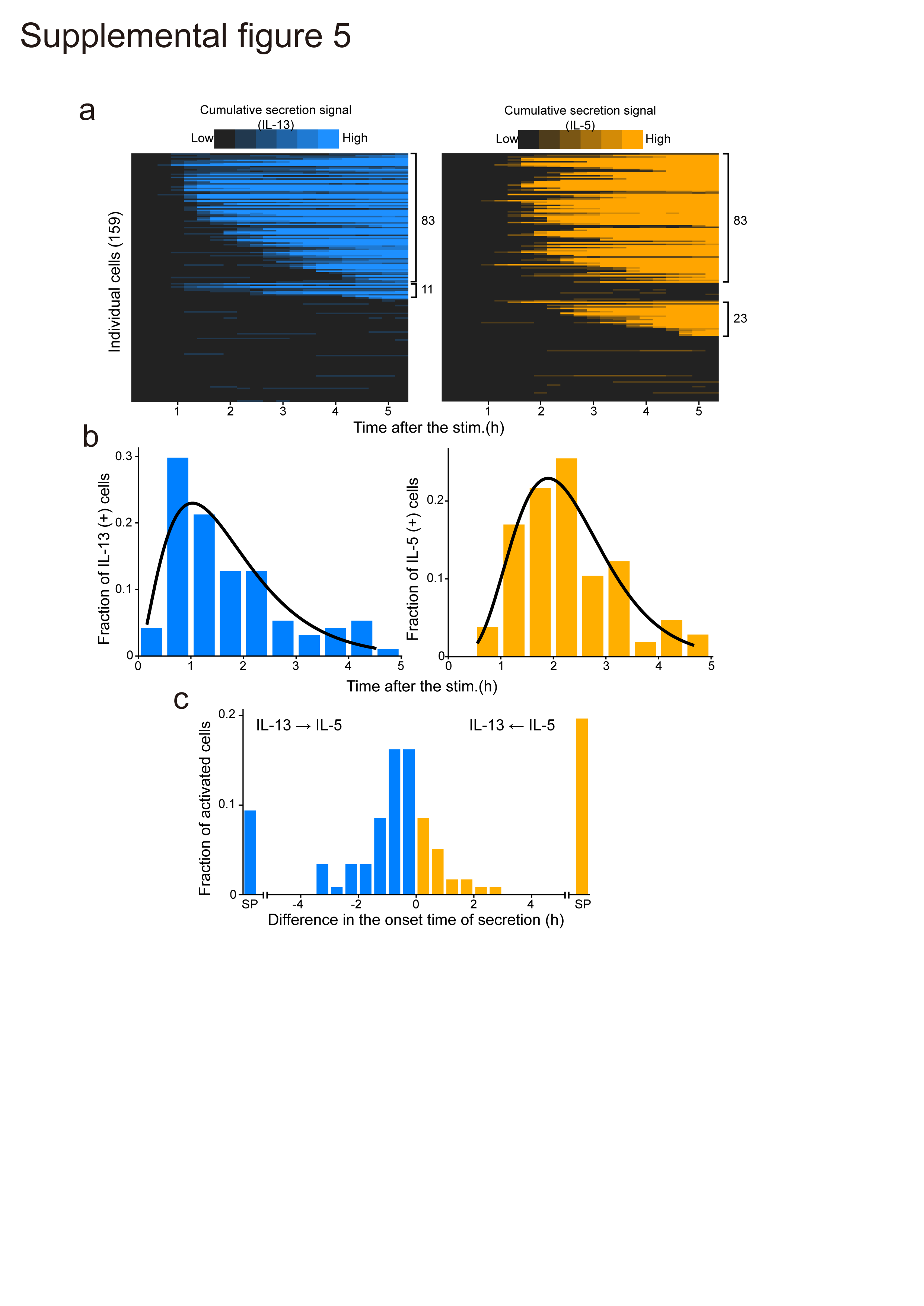
**Supplemental Fig. 5| Onset of IL-5 and IL-13 Secretion from Single-mILC2s**

(a) Simultaneous observation of IL-5 and IL-13 secretion activity, presented in heatmaps. 83 cells that secreted both cytokines, 11 cells secreted only IL-13, and 23 cells secreted only IL-5, with cells sorted in order of earliest onset of IL-13 secretion. (b) Histograms of IL-13 or IL-5 secretion onset. (c) Deviation in the onset time of secretion of IL-5 and IL-13 from the same cells. The onset of IL-13 secretion precedes that of IL-5 by 30 minutes.


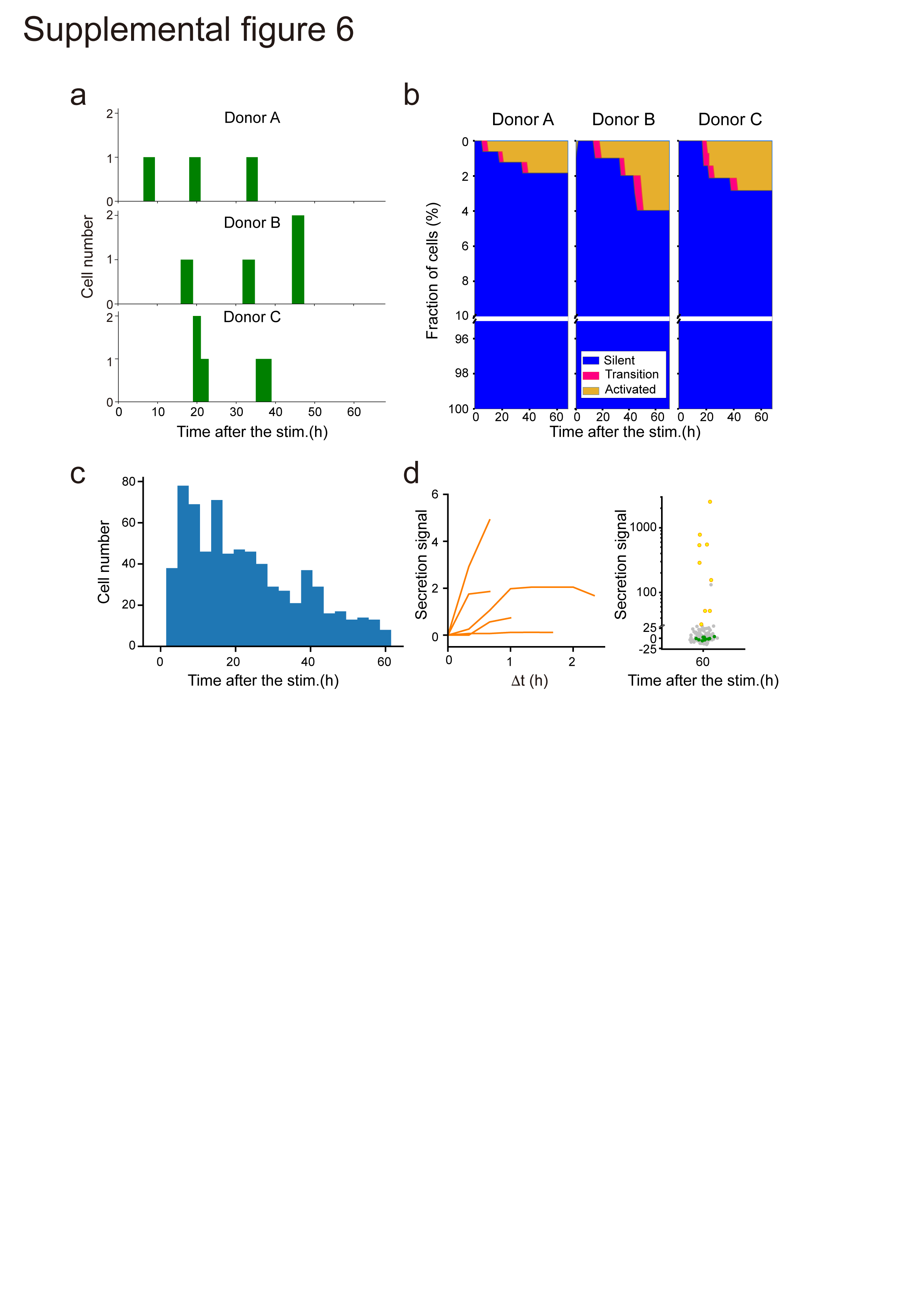


**Supplementary Fig. 6| Analysis of IL-13 Secretion in Stimulated Human ILC2s.**

(a) Histogram of the onset of IL-13 secretion in single ILC2s from three individuals, obtained independently of the real-time recovery experiment. (b) Changes in the percentage of cells in the pre-activation, transition state (Δt<3 hours), and activated states calculated from the secretion onset data in (a). (c) Distribution of IL-13 secretion onset obtained from data in a previously published paper^33^. (d) IL-13 signal traces of human ILC2s recovered in real-time (left) and signal intensity distribution of human ILC2s 60 hours after stimulation (right). The signal intensities of cells recovered as activated cells are shown in yellow, and those of cells collected as silent are shown in green.


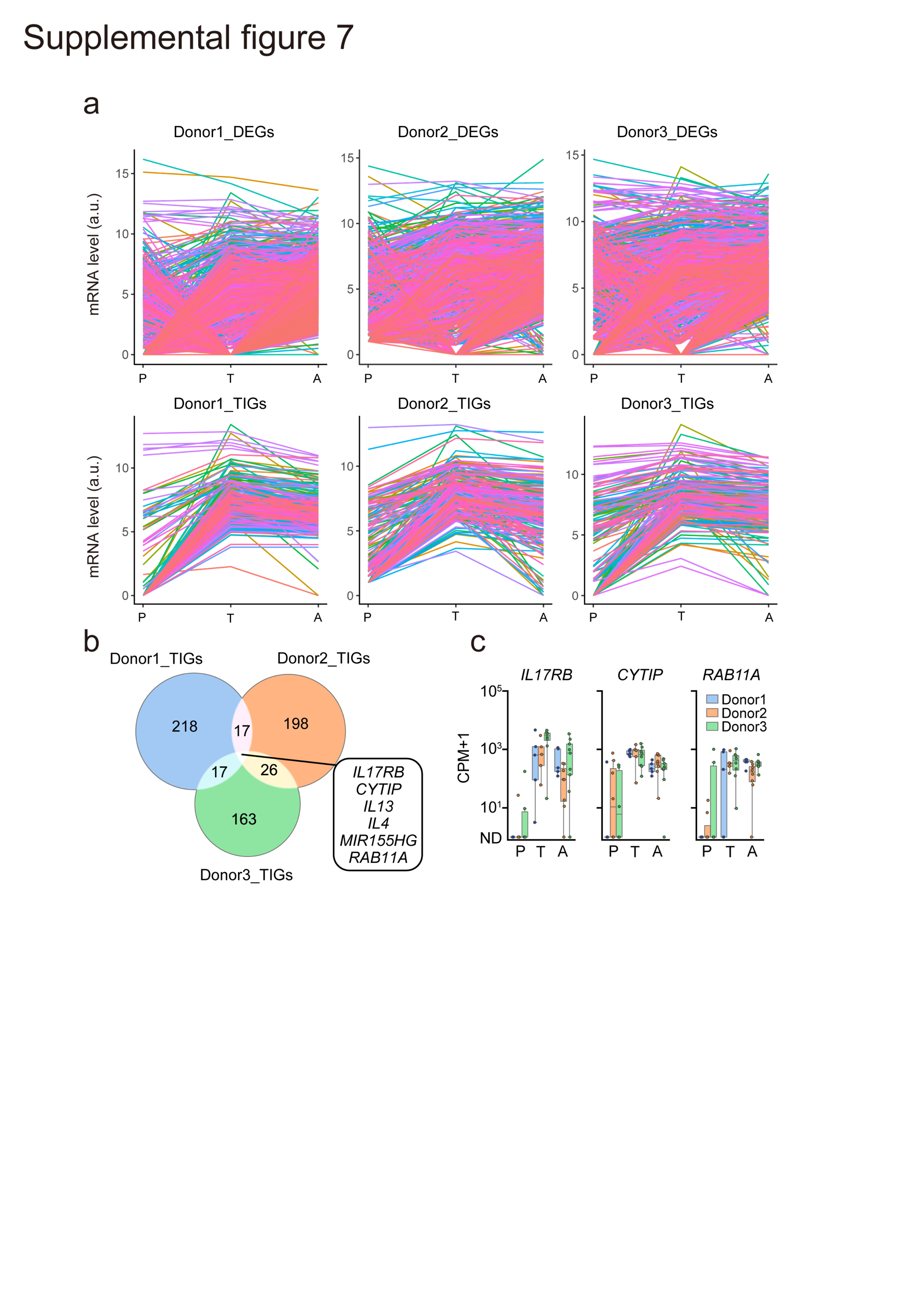
**Supplementary Fig. 7| TIG Detection of hILC2s.**(a) The relative expression levels of differentially expressed genes (DEGs) that exhibited variation among the three cellular states (top) and genes that displayed transiently induced gene (TIG)-like variation among them (bottom). P, pre-activation; T, transition state; A, activation. (b) A Venn diagram illustrates the overlap of TIGs among the three donors. (c) Box plots illustrating the expression levels of overlapping TIGs other than those shown in Fig. 4c. Each colour represents a different specimen. The centre line, box limits, whiskers, and dots indicate the median, upper and lower quartiles, 1.5x interquartile range and individual values, respectively. ND: not detected.


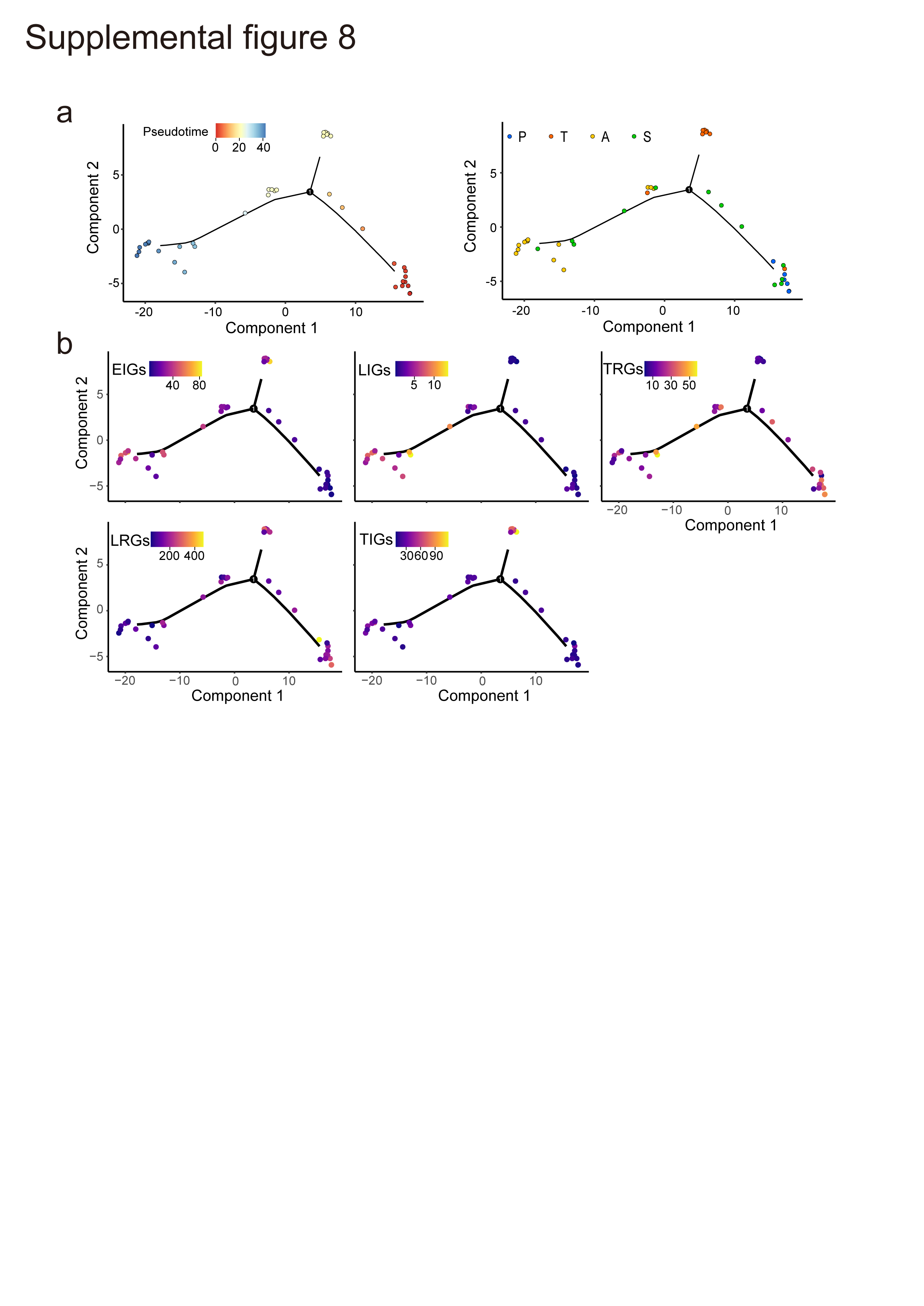


**Supplementary Fig. 8| Pseudo-time Analysis of hILC2 Activation Process**(a) and (b) depict the trajectory inference results obtained using Monocle. (a) The pseudo-time (left) and the activation states indexed by the TDCSS technique (right) in color. (b) The average expression levels of gene groups classified into five expression transition patterns by the TDCSS technique in color. Notably, LIGs and TIGs are highly expressed at the respective node ends following branching.

**
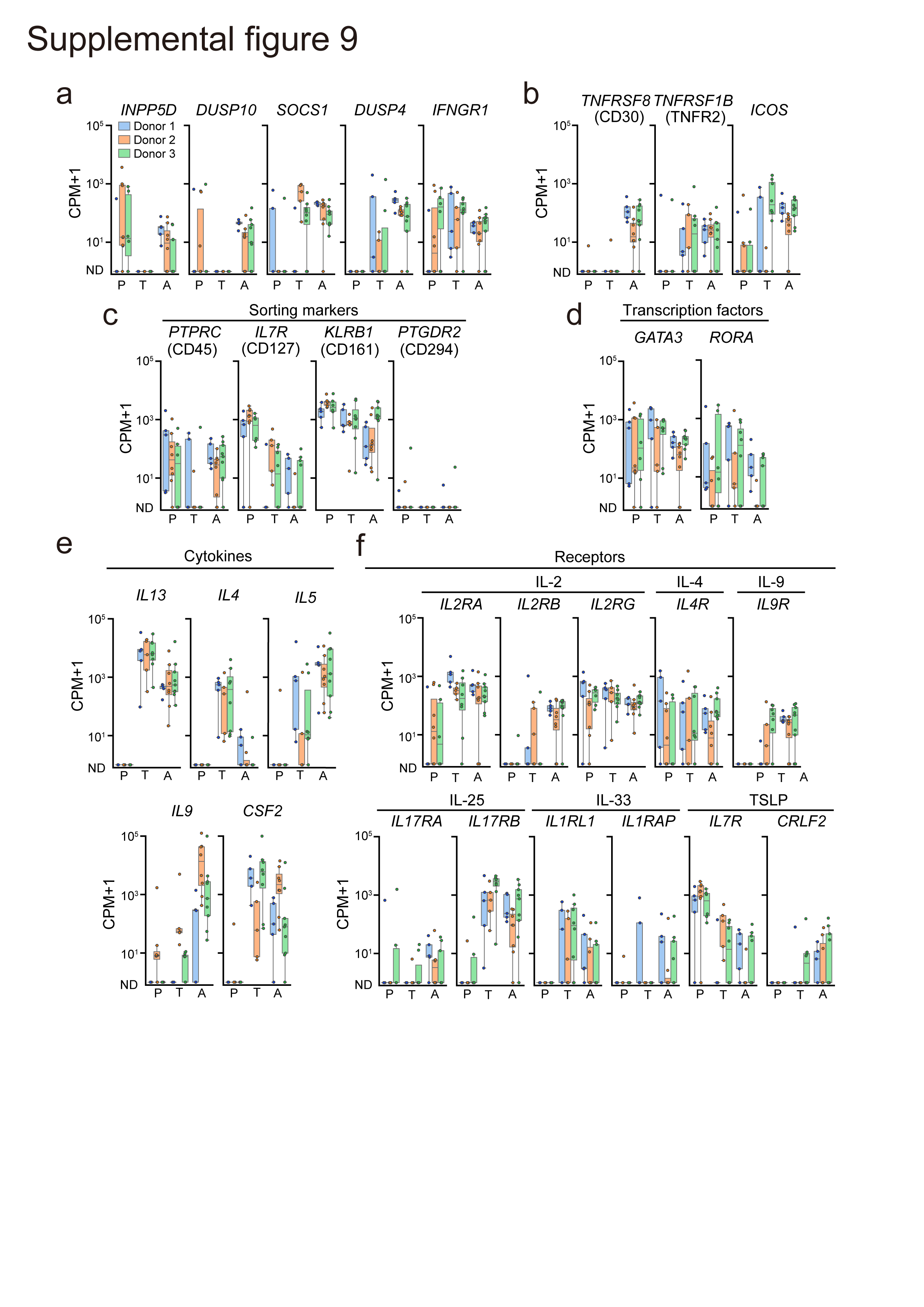
**

**Supplementary Fig. 9| Expression levels of individual genes associated with ILC2s.**

(a-f) Box plot illustrating the mRNA-seq data-derived expression levels of individual genes at each activation states; P, pre-activation; T, transition state; A, activated. Each colour represents a different specimen. The centre line, box limits, whiskers, and dots indicate the median, upper and lower quartiles, 1.5x interquartile range and individual cells, respectively. ND: not detected. (a) Genes encoding proteins known to inhibit signal pathways associated with ILC2 activation. (b) Genes encoding proteins identified as surface markers for CRTH2(-) IL7Rα (-) type 2 cytokine-producing ILCs. (c) Genes encoding proteins utilized as sorting markers for hILC2. (d) Genes encoding proteins considered to be master regulators of ILC2. (e) Genes encoding cytokines known to be produced by ILC2. (f) Genes encoding receptors for ILC2-activating cytokines.

**Abbreviations**

ILC2: Group 2 innate lymphoid cell

TDCSS: Time-Dependent Cell-State Selection

LCI: Live-Cell Imaging

LCI-S: Live-Cell Imaging of Secretion activity

IL: Interleukin

mILC2: mouse ILC2

hILC2: human ILC2

RNA: Ribonucleic Acid

mRNA: messenger RNA

PCR: Polymerase Chain Reaction

qRT-PCR: quantitative Reverse Transcription PCR

RNA-seq: RNA sequencing

DEG: Differential Expression Gene

GO: Gene Ontology

IFN-γ: Interferon gamma
