# Supplementary material for: Time-dependent cell-state selection identifies transiently expressed genes regulating ILC2 activation
Source: Commun Biol. 2023 Sep 6;6:915. doi: 10.1038/s42003-023-05297-w (PMC10482971; doi:10.1038/s42003-023-05297-w)
Supplement: Supplementary file 2 — Description of Additional Supplementary Files [file 42003_2023_5297_MOESM2_ESM.pdf]

Description of Additional Supplementary Files

File name: Supplementary Data 1
Description: Results of RNA-seq analysis of mILC2 recovered using IL-13 as an index (related to Figure 3). The first 65 columns are the RNA-seq count data of single-mILC2s. The values were log2 (Count Per Million + 1). At the "anova_p_val" column, the p-value of ANOVA among pre-stim. cells, dt0.5h cells, LA cells and LS cells are listed. At the "kmeans_cluster#" column, the results of kMeans clustering of genes with ANOVA pvalues less than 0.05 are listed. At the "Class of gene expression transition" column, the results of re-categorizing based on the variation patterns of the genes. The columns behind them show the relative gene expression levels in each cell state in Z-score.

File name: Supplementary Data 2
Description: Results of GO clustering analysis of mILC2 recovered using IL-13 as an index (related to Figure 3). We analyzed GO of four groups of differentially expressed genes (Pre-stim. < Activated, Pre-stim. < Silent, Silent < Activated and TIGs) by the Metascape. The "Annotation" sheet includes each gene's group, their converted gene ID, annotation, and membership. The "Enrichment" sheet includes enriched GO terms and their statistics.

File name: Supplementary Data 3
Description: Results of RNA-seq analysis of mILC2 recovered using IL-5 as an index (related to Suppl. Figure 4). The first 65 columns are the RNA-seq count data of single-mILC2s. The values were log2 (Count Per Million + 1). At the "anova_p_val" column, the p-value of ANOVA among prestim. cells, dt0.5h cells, LA cells and LS cells are listed. At the "k-means_cluster#" column, the results of k-Means clustering of genes with ANOVA p-values less than 0.05 are listed. At the "Class of gene expression transition" column, the results of recategorizing based on the variation patterns of the genes. The columns behind them show the relative gene expression levels in each cell state in Z-score.

File name: Supplementary Data4
Description: List of overlapping genes in TIGs obtained using IL-13 and IL-5 as indexes.

File name: Supplementary Data 5
Description: Results of GO clustering analysis of IL-13 TIGs and IL-5 TIGs (related to Suppl. Figure 4). We analyzed GO of IL-13 TIGs and IL-5 TIGs by the Metascape. The "Annotation" sheet includes each gene's group, their converted gene ID, annotation, and membership. The "Enrichment" sheet includes enriched GO terms and their statistics.

File name: Supplementary Data 6
Description: RNA-seq Count data of hILC2 (related to Figure 4). The first 59 columns are the RNA-seq count data of hILC2s. Column names represent the following sample information: D1: Donor 1, D2: Donor 2; D3: Donor 3; P: Pre-simt, T: Transition state, A: Activated. The values were log2 (Count Per Million + 1). The next nine columns show the mean values of CPM. At the "anova_p_val" column, the p-value of ANOVA among Prestim. cells, Transition cells and Activated cells are listed. At the "k-means_cluster#" column, the results of k-Means clustering of genes with ANOVA p-values less than 0.01 are listed. At the "Class of gene expression transition" column, the results of recategorizing based on the variation patterns of the genes. The columns behind them show the relative gene expression levels in each cell state in Z-score.

File name: Supplementary Data 7
Description: Source data used to generate the graphs and charts in the main figures. The label of each sheet represents the title of the corresponding figure.
