# Supplementary material for: Time-dependent cell-state selection identifies transiently expressed genes regulating ILC2 activation
Source: Commun Biol. 2023 Sep 6;6:915. doi: 10.1038/s42003-023-05297-w (PMC10482971; doi:10.1038/s42003-023-05297-w)
Supplement: Supplementary file 10 — Reporting Summary [file 42003_2023_5297_MOESM10_ESM.pdf]

nature portfolio

Corresponding author(s): Yoshitaka Shirasaki, Sotaro Uemura

Last updated by author(s): Jul 27, 2023

Reporting Summary

Nature Portfolio wishes to improve the reproducibility of the work that we publish. This form provides structure for consistency and transparency in reporting. For further information on Nature Portfolio policies, see our Editorial Policies and the Editorial Policy Checklist.

Statistics

For all statistical analyses, confirm that the following items are present in the figure legend, table legend, main text, or Methods section.

n/a	Confirmed	
☐	☒	The exact sample size (*n*) for each experimental group/condition, given as a discrete number and unit of measurement
☐	☒	A statement on whether measurements were taken from distinct samples or whether the same sample was measured repeatedly
☐	☒	The statistical test(s) used AND whether they are one- or two-sided<br>*Only common tests should be described solely by name; describe more complex techniques in the Methods section.*
☐	☒	A description of all covariates tested
☐	☒	A description of any assumptions or corrections, such as tests of normality and adjustment for multiple comparisons
☐	☒	A full description of the statistical parameters including central tendency (e.g. means) or other basic estimates (e.g. regression coefficient) AND variation (e.g. standard deviation) or associated estimates of uncertainty (e.g. confidence intervals)
☐	☒	For null hypothesis testing, the test statistic (e.g. *F*, *t*, *r*) with confidence intervals, effect sizes, degrees of freedom and *P* value noted<br>*Give P values as exact values whenever suitable.*
☐	☒	For Bayesian analysis, information on the choice of priors and Markov chain Monte Carlo settings
☐	☒	For hierarchical and complex designs, identification of the appropriate level for tests and full reporting of outcomes
☒	☐	Estimates of effect sizes (e.g. Cohen's *d*, Pearson's *r*), indicating how they were calculated

Our web collection on statistics for biologists contains articles on many of the points above.

Software and code

Policy information about availability of computer code

Data collection	NIS-Elements AR (ver.4.50.00, Nikon)
Data analysis	For the real-time analysis of acquired data in TDCSS, we used custom software deposited in GitHub.<br>For statistical test and clustering, we used Python3.

For manuscripts utilizing custom algorithms or software that are central to the research but not yet described in published literature, software must be made available to editors and reviewers. We strongly encourage code deposition in a community repository (e.g. GitHub). See the Nature Portfolio guidelines for submitting code & software for further information.

Data

Policy information about availability of data

All manuscripts must include a data availability statement. This statement should provide the following information, where applicable:

- Accession codes, unique identifiers, or web links for publicly available datasets
- A description of any restrictions on data availability
- For clinical datasets or third party data, please ensure that the statement adheres to our policy

RNA-sequencing data will be available through the GEO database. Source Data for Figs. 2–4 and suppl. Figs. 1–9 are provided with the paper. Other datasets generated during and/or analyzed during the current study are available from the corresponding authors on reasonable request.

Research involving human participants, their data, or biological material

Policy information about studies with human participants or human data. See also policy information about sex, gender (identity/presentation), and sexual orientation and race, ethnicity and racism.

Reporting on sex and gender	In this manuscript, our main objective was to demonstrate a novel approach to detect activation of ILC2s at the single cell level and to study its gene expression status. Therefore, we did not conduct a sex and gender-based study.
Reporting on race, ethnicity, or other socially relevant groupings	In this manuscript, our main objective was to demonstrate a novel approach to detect activation of ILC2s at the single cell level and to study its gene expression status. Therefore, we did not conduct a study on race, ethnicity, or other socially relevant groupings
Population characteristics	Blood samples were corrected from three healthy men in their 30s.
Recruitment	Samples were obtained from healthy volunteers at Keio University School of Medicine.
Ethics oversight	*Identify the organization(s) that approved the study protocol.*

Note that full information on the approval of the study protocol must also be provided in the manuscript.

Field-specific reporting

Please select the one below that is the best fit for your research. If you are not sure, read the appropriate sections before making your selection.

☒ Life sciences ☐ Behavioural & social sciences ☐ Ecological, evolutionary & environmental sciences

For a reference copy of the document with all sections, see nature.com/documents/nr-reporting-summary-flat.pdf

Life sciences study design

All studies must disclose on these points even when the disclosure is negative.

Sample size	No statistical methods were used to determine sample size.<br>In the scRNA-seq experiments using mILC2s, we collected 10 (Pre-stimulation) or 11 (others) single-cells in each activation stage . In total, we collected about 60 cells.<br>In the scRNA-seq experiments using hILC2s, we collected 5 to 8 cells in each activation stage from three individuals.
Data exclusions	In the scRNA-seq experiments, we exclude samples in which library preparation were failed. We checked the library quality of each sample using BioAnalyzer and exclude samples whose cDNA was degraded or not amplified.
Replication	In the mILC2 experiments, we replicated four times to collect samples for RNA-seq. In the hILC2 experiments, we replicated three times using different individual samples.
Randomization	In the mILC2 experiments, we used pooled ILC2s from 20 animals.<br>In the hILC2 experiments, we randomly selected volunteers and used all data acquired from them.
Blinding	All human specimens used in this study were de-identified.

Reporting for specific materials, systems and methods

We require information from authors about some types of materials, experimental systems and methods used in many studies. Here, indicate whether each material, system or method listed is relevant to your study. If you are not sure if a list item applies to your research, read the appropriate section before selecting a response.

Materials & experimental systems

n/a	Involved in the study	
☐	☒	Antibodies
☒	☐	Eukaryotic cell lines
☒	☐	Palaeontology and archaeology
☐	☒	Animals and other organisms
☒	☐	Clinical data
☒	☐	Dual use research of concern
☒	☐	Plants

Methods

n/a	Involved in the study	
☒	☐	ChIP-seq
☐	☒	Flow cytometry
☒	☐	MRI-based neuroimaging

Antibodies

Antibodies used	For cytokine secretion imaging by LCI-S , we used anti-human IL-13 (MAB213 and BAF 213) and IL-4 (MAB604 and BAF204), anti-mouse IL-13 (MAB413 and BAF413) and IL-5 (MAB405 and BAM705) antibodies (R&D Systems, Minneapolis, MN, USA).<br>For cell sorting of human ILC2s, we used lineage antibody cocktails of human CD3 (UCHT1), CD14 (HCD14), CD16 (3G8), CD19 (HIB19), CD20 (2H7), CD56 (HCD56), PE-conjugated anti-human CD127 (A019D5), PE/Cy7-conjugated anti-human CD161 (HP-3G10) antibodies (BioLegend, San Diego, CA, USA), and Alexa Fluor 647-conjugated anti-human CRTH2 (BM16) antibody (BD Biosciences, San Jose, CA, USA).<br>For cell sorting of mouse ILC2s, we used antibodies described in [Moro, K., Ealey, K., Kabata, H. et al. Isolation and analysis of group 2 innate lymphoid cells in mice. Nat Protoc 10, 792–806 (2015). https://doi.org/10.1038/nprot.2015.047].
Validation	All antibodies used have been obtained from commercial vendors. Validation information can be found on the respective Vendor websites.

Animals and other research organisms

Policy information about studies involving animals; ARRIVE guidelines recommended for reporting animal research, and Sex and Gender in Research

Laboratory animals	Wildtype C57BL/6 retired female breeder mice
Wild animals	The study did not involve wild animals.
Reporting on sex	In this manuscript, our main objective was to demonstrate a novel approach to detect activation of ILC2s at the single cell level and to study its gene expression status. Therefore, we did not conduct a sex and gender-based study.
Field-collected samples	The study did not involve samples collected from the field.
Ethics oversight	All experiments were approved by the Animal Care and Use Committee of RIKEN and performed according to institutional guidelines.

Note that full information on the approval of the study protocol must also be provided in the manuscript.

Flow Cytometry

Plots

Confirm that:

- [ ] The axis labels state the marker and fluorochrome used (e.g. CD4-FITC).
- [ ] The axis scales are clearly visible. Include numbers along axes only for bottom left plot of group (a 'group' is an analysis of identical markers).
- [ ] All plots are contour plots with outliers or pseudocolor plots.
- [ ] A numerical value for number of cells or percentage (with statistics) is provided.

Methodology

Sample preparation	We obtained the mouse ILC2s as described in [Moro, K., Ealey, K., Kabata, H. et al. Isolation and analysis of group 2 innate lymphoid cells in mice. Nat Protoc 10, 792–806 (2015). https://doi.org/10.1038/nprot.2015.047].<br>For human ILC2, we obtained peripheral blood mononuclear cells (PBMCs) from 20 mL of peripheral blood of volunteers using lymphocyte separation medium Lymphoprep™ (Cosmo Bio) according to the manufacture's protocols. Then human ILC2s were sorted from PBMCs by flow cytometry.
Instrument	Mouse ILC2s were isolated by FACSAria™ IIIu (BD FACS Diva Software Version 9.0.1) (BD Bioscience).<br>Human ILC2s were isolated by MoFlo™ XDP (Beckman Coulter).
Software	Data were analysed by FlowJo software (v10.6.1 or v10.7.1) (BD Bioscience).
Cell population abundance	Gating strategies was confirmed by backgating, and post-sort fractions were not analyzed for checking the purity.

Gating strategy

Gating strategies for mouse ILC2s are reported in method.
FSC-A/SSC-A gates were used to exclude cell debris.
FSC-H/FSC-W and SSC-H/SSC-W, or FSC-A/FSC-H gates were used to exclude doublet cells.
Mouse ILC2s were defined as Lineage-(CD3ε, CD4, CD8α, CD11c, FcεRIα, NK1.1, CD19, TER119, F4/80, Gr-1) c-Kit+Sca-1+ cells.

Gating strategies for human ILC2s are as below.
FSC-A/SSC-A gates were used to exclude cell debris.
PI low gate were used to exclude damaged cells.
human ILC2s were defined as Lineage-(CD3, CD14, CD16, CD19, CD20 and CD56) CD45+ CD127+ CRTH2+ CD161+ cells.

☐ Tick this box to confirm that a figure exemplifying the gating strategy is provided in the Supplementary Information.
